# Effects of β-Lactam Antibiotics and Fluoroquinolones on Human Gut Microbiota in Relation to *Clostridium difficile* Associated Diarrhea

**DOI:** 10.1371/journal.pone.0089417

**Published:** 2014-02-28

**Authors:** Henrik Knecht, Sven C. Neulinger, Femke Anouska Heinsen, Carolin Knecht, Anke Schilhabel, Ruth A. Schmitz, Alexandra Zimmermann, Vitor Martins dos Santos, Manuel Ferrer, Philip C. Rosenstiel, Stefan Schreiber, Anette K. Friedrichs, Stephan J. Ott

**Affiliations:** 1 Institute of Clinical Molecular Biology (IKMB), Christian-Albrechts-University (CAU), Kiel, Germany; 2 Institute for General Microbiology (IFAM), Christian-Albrechts-University (CAU), Kiel, Germany; 3 Institute of Medical Informatics and Statistics (IMIS), Christian-Albrechts-University (CAU), Kiel, Germany; 4 Systems and Synthetic Biology, Wageningen University, Wageningen, The Netherlands; 5 LifeGlimmer GmbH, Berlin, Germany; 6 Laboratory of Enzyme Discovery, CSIC - Institute of Catalysis, Madrid, Spain; 7 Department of Internal Medicine I, University Hospital Schleswig-Holstein (UKSH), Kiel, Germany; Charité-University Medicine Berlin, Germany

## Abstract

*Clostridium difficile* infections are an emerging health problem in the modern hospital environment. Severe alterations of the gut microbiome with loss of resistance to colonization against *C. difficile* are thought to be the major trigger, but there is no clear concept of how *C. difficile* infection evolves and which microbiological factors are involved. We sequenced 16S rRNA amplicons generated from DNA and RNA/cDNA of fecal samples from three groups of individuals by FLX technology: (i) healthy controls (no antibiotic therapy); (ii) individuals receiving antibiotic therapy (Ampicillin/Sulbactam, cephalosporins, and fluoroquinolones with subsequent development of *C. difficile* infection or (iii) individuals receiving antibiotic therapy without *C. difficile* infection. We compared the effects of the three different antibiotic classes on the intestinal microbiome and the effects of alterations of the gut microbiome on *C. difficile* infection at the DNA (total microbiota) and rRNA (potentially active) levels. A comparison of antibiotic classes showed significant differences at DNA level, but not at RNA level. Among individuals that developed or did not develop a *C. difficile* infection under antibiotics we found no significant differences. We identified single species that were up- or down regulated in individuals receiving antibiotics who developed the infection compared to non-infected individuals. We found no significant differences in the global composition of the transcriptionally active gut microbiome associated with *C. difficile* infections. We suggest that up- and down regulation of specific bacterial species may be involved in colonization resistance against *C. difficile* providing a potential therapeutic approach through specific manipulation of the intestinal microbiome.

## Introduction

*Clostridium difficile-*associated diarrhea (CDAD) is caused by *C. difficile* toxins A and B and represents a serious burden for the clinical environment and the health care system [1]. Although the natural intestinal microbiota of healthy individuals can prevent colonization by *C. difficile* [2], [3], the incidence of *C. difficile* infections has increased in the last decade [4]. Although approximately 5% of the adult population is colonized by *C. difficile* without showing any symptoms of diarrhea, the colonization rate increases up to 20% in hospitalized individuals [5]. Other factors such as advanced age, parenteral nutrition, renal insufficiency, previous hospitalizations, treatment with proton pump inhibitors and previous antibiotic (AB) treatment increase the risk of developing CDAD [6]–[8]. Several studies have shown an association between AB treatments such as Ampicillin, cephalosporins and fluoroquinolones and an increased risk of developing CDAD [9], [10]. AB treatment can lead to AB-induced dysbiosis of the host-associated microbiome, with the potential absence of important gut microbial commensal species and an indirect break of mutualistic interactions [11], [12]. This dysbiosis may result in the unlimited expansion of *C. difficile* [13], [14]. Only a few studies have investigated the effect of AB on the dynamic changes of the intestinal microbiome in relation to the development of CDAD in humans. In additions to an increase of *C. difficile*, shifts within Bacteroidetes, *C. coccoides*, *Eubacterium rectale*, *Ruminococcus gnavus* and *C. nexile* have been shown [15]. A marked decrease in the microbial alpha diversity (the variety of organisms inhabiting a defined habitat) is likely responsible for facilitating intestinal colonization with *C. difficile* as has been observed by temperature gradient gel electrophoresis (TGGE) and bacterial clone libraries [14]–[16]. Manges *et al*. illustrated that low levels of Bacteroidetes are significantly associated with CDAD [14] and other data suggest that the absence of Bacteroidetes results in chronically relapsing *C. difficile* diarrhea [17]. A study investigating *C. difficile-*colonized infants revealed that low Firmicutes and Bacteroidetes ratios and an increase in facultative anaerobes facilitate colonization with *C. difficile* [18]. No previous studies have highlighted the specific role of the species present in the gut microbiota (indicated by the amounts of the 16S rRNA genes generated from DNA) and particularly the metabolically active species (indicated by the amounts of the 16S rRNA amplicons generated from cDNA) in individuals with CDAD due to antibiotic therapy [19].

We hypothesize that the gut microbial composition of individuals who develop CDAD is different from that of CDAD-negative individuals, in terms of the total and the active microbiota as determined by alpha and beta diversity measures. We further postulate that the alteration of the gut microbial composition is similar for different ABs (fluoroquinolones, cephalosporins and Ampicillin/Sulbactam).

## Results

We compared the total microbiota and the microbiota with protein synthesis potential (potentially active) [19] of individuals developing CDAD to those not developing CDAD during the course of AB treatment with cephalosporins or Ampicillin/Sulbactam (n = 5 each). This investigation was conducted by analysis of the V1–V2 variable regions of the bacterial 16S rRNA gene amplified from DNA or cDNA generated from RNA. We also investigated alterations of the gut microbiota composition induced by AB treatment with different AB classes, including fluoroquinolones, cephalosporins and Ampicillin/Sulbactam (n = 5 each). Control cases (n = 18) who did not receive antibiotic therapy were also included in the study.

Pyrosequencing yielded 524,582 sequences across all samples after preprocessing and a quality check. Sequences have been deposited in MG-RAST (http://metagenomics.anl.gov) under accession numbers 4546490.3 to 4546560.3. Random subsampling produced 96,387 sequences (1,083 reads for each sample) for further analysis. The sequences were binned at the 97% similarity level into a total of 4,991 operational taxonomic units (OTUs), with 2,763 and 3,175 OTUs at the DNA and RNA levels, respectively, with an avg. length of 355 bp in both cases.

### Alpha diversity

**Figure 1:**
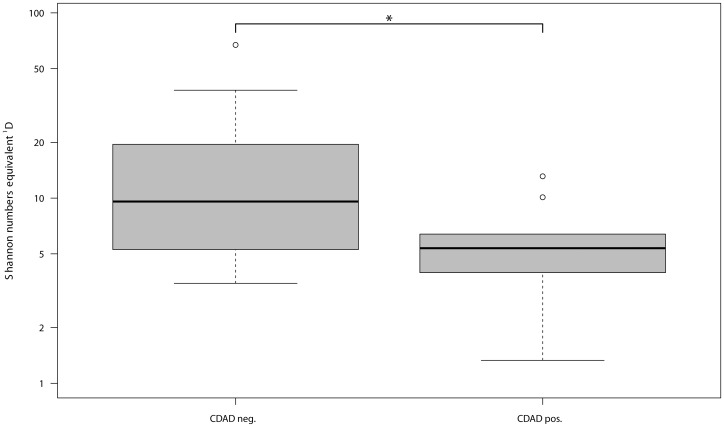
Distribution of Shannon number equivalents at DNA level concerning CDAD positive and CDAD negative individuals.

**Figure 2:**
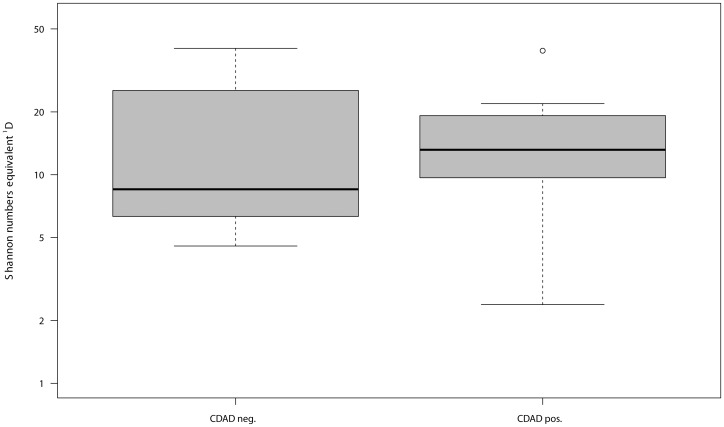
Distribution of Shannon number equivalents at RNA level concerning CDAD positive and CDAD negative individuals.

As illustrated in Figure 1 and 2, significant differences in bacterial alpha diversity at the DNA (P *_C. difficile_*_ DNA_ = 0.05) but not at the RNA level (P *_C. difficile_*_ RNA_ = 0.86) were found between individuals with CDAD and the individuals without CDAD during AB therapy. Differences between the effects of Ampicillin/Sulbactam and cephalosporin treatments were not significant at either level (P _AB DNA_ = 0.90, P _AB RNA_ = 0.20). Consequentially, these antibiotics were treated as one group, named the ß-lactams (Ampicillin/Sulbactam/cephalosporins) in comparison to the fluoroquinolones and controls. Pair-wise differences regarding the bacterial alpha diversity of the healthy individuals and individuals treated with the ß-lactam antibiotics or fluoroquinolones were not statistically significant at the DNA level. At the RNA level the difference between healthy controls and individuals treated with ß-lactam antibiotics reached statistical significance (Figure S1) (P _Contr-ß-lactam RNA_ = 0.01).

### Beta diversity

Redundancy analyses (RDA) with subsequent pair-wise comparisons were used to assess the differences in total and potentially active OTUs of the gut microbial community in the individuals with and without CDAD development during AB treatment. No significant difference was found in total and potentially active gut microbiota of individuals with CDAD compared to individuals without CDAD.

**Figure 3:**
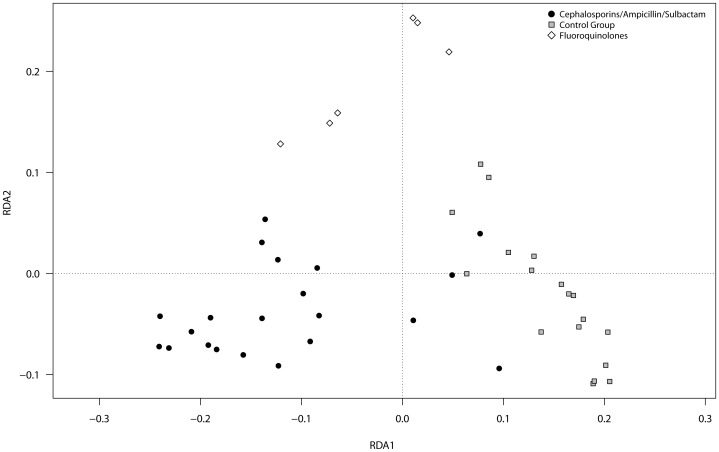
Graphical representation (distance plots) of the redundancy analysis (RDA) model of Hellinger-transformed OTU abundances. The model illustrate the relationship of gut microbes of healthy controls, individuals treated with ß-lactam antibiotic (Cephalosporins/Ampicillin/Sulbactam) and individuals treated with fluoroquinolones at DNA level.

Individuals treated with Ampicillin/Sulbactam or cephalosporins showed no significant differences in total and potentially active gut microbiota. These groups were further treated as one group (cephalosporins/Ampicillin/Sulbactam (ß-lactam)). Graphical representations of the RDA models are shown in Figure 3 (DNA) and 4 (RNA). The gut microbiota of individuals treated with ß-lactam were significantly different from those treated with fluoroquinolones at the DNA level (P_DNA_<0.01), but not at the RNA level (P_RNA_ = 0.26). Healthy controls harbored gut microbiota significantly different compared to those of individuals treated with either ß-lactam antibiotics or fluoroquinolones at both levels (P_ß-Lactam DNA_<0.01, P_fluoroquinolones DNA_<0.01, P_ß-Lactam RNA_<0.01, P_fluoroquinolones RNA_<0.01).

**Figure 4:**
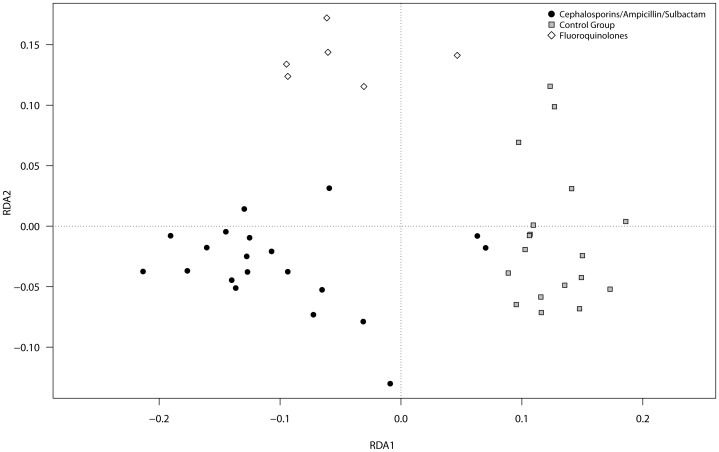
Graphical representation (distance plots) of the redundancy analysis (RDA) model of Hellinger-transformed OTU abundances. The model illustrate the relationship of gut microbes of healthy controls, individuals treated with ß-lactam antibiotic (Cephalosporins/Ampicillin/Sulbactam) and individuals treated with fluoroquinolones at RNA level.

### Bacterial distribution in fecal samples (OTU-independent)

As shown in Figures S2 and S3, fluctuations among the relative amounts of bacterial phyla and families were observed at the DNA and RNA levels. Firmicutes were the dominant phylum in all control and AB samples.

Compared to controls, treatment with ß-lactam antibiotics resulted in a decrease in Firmicutes and increase of Bacteroidetes at the DNA and RNA levels. This decrease did not occur with fluoroquinolone treatment. The relative amount of Proteobacteria increased at DNA level (mean increase from 0,65%_DNA-controls_ to 15%_DNA-ß-lactam_, 1,85%_DNA-fluoroquinolones)_ and RNA level (mean increase from 12,48%_RNA-controls_ to 22,5%_RNA-ß-lactam_, 17,24%_RNA-fluoroquinolones_) due to treatment with ß-lactam antibiotics and fluoroquinolones in all individuals. At the bacterial family level AB treatments resulted in an increase of Enterobacteriaceae (mean increase from 0,38%_DNA-controls_ to 15%_DNA-ß-lactam_, 1,7%_DNA-fluoroquinolones_) at DNA but not at RNA level. Furthermore, a marked decrease of Lachnospiraceae, compared to controls, at RNA level (mean decrease from 23%_RNA-controls_ to 6,6%_RNA-ß-lactam_, 15%_RNA-fluoroquinolones_) was observed. Thereby, reduction of Lachnospiraceae in individuals with CDAD (mean decrease from 23%_RNA-controls_ to 4,4%_RNA-ß-_lactam and 17%_DNA-controls_ to 4%_DNA-ß-lactam_) differed from individuals without CDAD (mean decrease from 23%_RNA-controls_ to 9%_RNA-ß-lactam_ and 17%_DNA-controls_ to 11%_DNA-ß-lactam_).

### Indicator taxa

Indicator OTUs (in analogy to indicator taxa) are OTUs predominant in a certain sample type while only irregularly found in a comparison sample type. Because no significant difference in gut microbial composition was observed among individuals treated with different ABs at the RNA level (fluoroquinolones compared to ß-lactam antibiotics), an indicator analysis was performed by comparing all AB samples treated as one group to the healthy controls at the RNA level. Indicator OTUs listed in Tables S1a–c were responsible for differences between controls and individuals treated with ß-lactam antibiotics and fluoroquinolones at DNA level (Table S1a) and between controls and ABs at the RNA level (Table S1b). Eighteen and 20 indicator OTUs were identified at the DNA and RNA levels, respectively. Eight OTUs at RNA level were indicators for healthy controls and showed homology to *Faecalibacterium prausnitzii*, *Coprococcus*, *Eubacterium*, Lachnospiraceae and *Clostridium.* Twelve OTUs were indicators for AB treatment, affiliated with *Blautia*, *Streptococcus parasanguinis*, *Clostridium ramnosum*, *Enterococcus lacti*, *Halomonas*, *Propionibacterium acnes and Staphylococcus epidermidis.* At DNA level six OTUs indicating healthy controls showed homology to *Faecalibacterium prausnitzii*, *Coprococcus* and *Eubacterium.* Ten OTUs indicating fluoroquinolone treatment were associated with *Blautia, Subdoligranulum, Adlercreutzia, Clostridium and Ruminococcus.* Two OTUs (*Enterobacter*, and *Enterococcus lacti*) indicated treatment with ß-lactam antibiotics.

No overall effect on the gut microbial composition was found in case CDAD. Rather an effect was found on single OTUs, as few microbial indicators could be identified as specific for CDAD during AB treatment. Table S1c shows the indicator OTUs found in individuals with and without CDAD at RNA and DNA levels; briefly, four indicator OTUs associated to individuals with CDAD (*Streptococcus*, *Enhydrobacter* and *Granulicatella* at RNA, and, as expected, *Clostridium difficile* at the DNA level) and two in non-CDAD individuals (*Adlercreutzia* and *Collinsella* at DNA level).

## Discussion

*C. difficile* infections can be provoked by intestinal detrimental events caused by AB-induced microbial imbalances and a loss of colonization resistance. As bacterial protein synthesis potential may indicate an important pathophysiological role, we examined additionally to DNA the amounts of 16S rRNA amplicons generated from cDNA in individuals receiving AB treatment. Although a few studies have investigated these imbalances based on AB treatment in the context of CDAD development in humans, we present the first study based on high-throughput sequencing technology that investigates the development of CDAD depending on the gut microbial profiles associated with specific AB therapy. As it was not possible to obtain larger sample sizes for each group we expected to recognize only large effects between the compared groups.

### Decreased alpha diversity in CDAD development at the DNA, but not at the RNA level

Alpha diversity expressed as effective OTU richness ^1^D (a.k.a. Shannon numbers equivalent) was used to assess differences in cumulative OTU abundance. ^1^D was consistently and significantly higher (by factor 2) in individuals without CDAD than in individuals with CDAD at the DNA level but not at the RNA level. Higher ^1^D is associated with higher species richness and/or evenness. Our results reveal that lower species richness at the DNA level might be indicative of colonization with *C. difficile*. These results at the DNA level are in agreement with the results of Chang *et al.*, who used bacterial clone libraries and observed that an overall collapse of bacterial alpha diversity (Shannon index) is responsible for *C. difficile* colonization [15]. Alpha diversity was not significantly different at the RNA level, indicating a rather stable potentially active microbiota. As the potentially active gut microbiota is more important to the host, these results may be more relevant. Results at RNA level contradict those at DNA level and support the assumption that the development of CDAD is not related to a collapse in alpha diversity of the gut microbiota, although AB therapy has decreased microbial α-diversity. It is possible that a reduction in the amounts of specific potentially active OTUs that may be involved in colonization resistance against *C. difficile* correlates with a loss of their functional role in the gut (i.e. the production of metabolites and antimicrobial substances), which could predispose individuals to *C. difficile* colonization.

### Development of CDAD is independent of gut microbial composition

The AB therapy used in this study affected the human gut microbiota and led to compositional shifts. Gut microbial composition showed no connection to CDAD development during AB treatment with ß-lactam antibiotics at DNA or RNA level. Because active gut bacterial diversity did not significantly change with CDAD, the loss of colonization resistance against *C. difficile* does not appear to be related to an overall collapse or overall reduction of the composition of the human gut microbiota during AB treatment. This trend was also shown by Reeves *et al.* in a murine model [20], which suggested that specific changes of single gut microbial members in the course of AB treatment may affect the susceptibility of the host to *C. difficile*. This finding is consistent with the clinical results of Blondeau *et al*. showing that the risk of CDAD development differs with different ABs based on their antimicrobial spectrum [21].

This study showed for the first time that development of CDAD is not dependent on the specific composition of the potentially active gut microbiota. The microbiota with protein synthesis potential are more essential for the host than the inactive microbiota because of factors such as metabolic turnover. Because no specific active gut microbial composition is associated with CDAD, the loss of colonization resistance against *C. difficile* is more likely to depend on the amounts of specific gut microbial members and their functional capacities rather than on the overall compositional level. In cases of CDAD a loss of the functional capacity of single species, possibly involving the production of metabolites such as short chain fatty acids (SCFA) or antimicrobial compounds, could allow the growth of *C. difficile*. Several investigators have reported that low concentrations of SCFA in the gut are associated with the presence of *C. difficile* [22], [23]. Reeves *et al.* showed that high levels of Lachnospiraceae contributed to colonization resistance against *C. difficile* or other pathogens in mice [20]. Members of Lachnospiraceae have the ability of ferment complex carbohydrates to SCFA and assist maintaining intestinal homeostasis [24]. In our study the gut microbiota of individuals with CDAD compared to CDAD negative individuals showed lower amounts of Lachnospiraceae as a result of AB treatment. Members of Lachnospiraceae were further identified as an indicator OTU at RNA level belonging to a healthy host. High or low levels of specific gut members such as Lachnospiraceae in relation to their functional role could play a central role in colonization resistance against *C. difficile* in humans. A study involving collection of fecal samples of individuals before and during CDAD development in the course of AB treatment could clarify the role of Lachnospiraceae and other specific gut members in humans with regard to colonization resistance against *C. difficile.*

### Different AB classes reduce the same beneficial bacteria and lead to an increase in opportunistic pathogens

We demonstrated that treatment with antibiotics such as Ampicillin/Sulbactam and cephalosporins (both ß-lactam antibiotic) affected the total gut microbiota in a similar manner. Treatment with fluoroquinolones affected the total gut microbiota differently from β-lactam treatment. The effects of Ampicillin/Sulbactam, cephalosporins and fluoroquinolones on the potentially active gut microbiota were indistinguishable. This finding led to the development of identical indicator OTUs at the RNA level for the different AB classes. Indicator OTUs associated with *Faecalibacterium prausnitzii* have been reported to be decreased due to AB treatment [25]. A decrease in *Faecalibacterium prausnitzii* as a result of AB treatment is important because this bacterium exhibits anti-inflammatory capacity and a reduction in the amount of this bacterium was shown to be associated with inflammatory diseases such as Crohn's disease (CD) or ulcerative colitis (UC) [2]–[27]. A reduction in potentially active *Coprococcus* was induced by both AB classes. This bacterium has been reported to be gut protective and beneficial for human health [28]–[30]. There was an increase in potentially active bacteria, including *Streptococcus parasanguinis*, *Clostridium rhamosum*, *Enterococcus lacti*, *Eggerthella lenta*, *Halomonas*, *Ralstonia*, and *Propionibacterium acnes* during AB treatment. The presence of *Streptococcus parasanguinis* was shown in previous studies to be associated with healthy oral microflora, and *Eggerthella lenta* has been reported to be a cause of UC [31], [32]. *Propionibacterium acnes* is a known opportunistic pathogen that normally does not harm its host. Low host resistance and high amounts of *P. acnes* have been shown to be involved in prostate inflammation, sarcoidosis and SAPHO (synovitis, acne, pustulosis, hyperostosis, osteitis). The increase of these potentially active opportunistic pathogens induced by AB medication may be important, especially in association with decreased host resistance. The observed higher amounts during AB therapy may be caused by AB resistance mechanisms, as it has been reported for *P. acnes* and *E. lenta* [33]–[35].

The effects of the ABs used in this study were similar at the RNA level. Some ABs target cell wall synthesis (cephalosporins and Ampicillin/Sulbactam), while others interfere with essential bacterial enzymes (fluoroquinolones) [36], [37]. This finding suggests that the most important gut microbiota members with protein synthesis potential are affected in similar ways independently of the AB class. The majority of previous studies investigated the gut microbiota during AB treatment at the level of total species (DNA). These studies observed different shifts for different ABs [38]–[41]. Our results support the previous findings at the DNA level but not at the RNA level. Similar effects of different ABs on the gut microbiota at the RNA level could explain why various ABs are associated with identical intestinal diseases such as AB-associated diarrhea.

### Conclusion

Employing high-throughput sequencing we showed that different AB classes (Ampicillin/Sulbactam, cephalosporins and fluoroquinolones) affect the gut microbiota similarly at the RNA level but differently at the DNA level. This finding explains why several different AB classes are associated with similar intestinal problems. CDAD development during AB therapy is not associated with a specific gut microbial composition at the RNA or DNA level. AB treatment affects the microbial composition and decreases the bacterial α-diversity, but no overall diversity collapse caused by AB treatment is responsible for the weak colonization resistance against *C. difficile*. CDAD development is a multifactorial pattern involving the interplay of gut microbial shifts and reduction of specific gut members and diversity caused by AB treatment which can lead to a lack of their functional integrity in colonization resistance against *C. difficile*. Future studies should investigate the development of CDAD and mechanisms of colonization resistance based on functional methods such as metatranscriptomics or metabolomics. Identification of single gut members contributing to colonization resistance may be helpful in determining the process of CDAD development in humans and may allow development of specific probiotics against CDAD. Furthermore, as we could not check for the type of *C.difficile* strains in infected patients, it would be interesting in future studies to characterize the type of *C.difficile* strains from gut samples of CDAD-positive individuals. This has the potential to correlate *C.diffi*cile strain types with specific changes of the gut microbiota as well as severity of clinical symptoms.

## Materials and Methods

### Study design

Study volunteers provided fecal samples and were divided into three different groups. Feces, collected from eighteen healthy individuals, were used as controls in this study (named “Controls”). Furthermore, fecal samples were obtained from hospitalized individuals five days after starting a therapy with one of the following antibiotic classes classified as fluoroquinolones (n = 5), cephalosporins) (n = 5) or Ampicillin/Sulbactam (penicillins) (n = 5) (named “AB-controls”) in the Department for Internal Medicine at the University Hospital Schleswig-Holstein, Campus Kiel (UK-SH). Only samples obtained from individuals who did not develop CDAD during and up to 3 months after the antibiotic therapy were included in the group of AB controls. The third group (named “*C. difficile*”) consisted of fecal samples collected from hospitalized individuals immediately after developing CDAD during therapy with cephalosporins or Ampicillin/Sulbactam (each with n = 5) (overview of study participants in Table S2). Further criteria for participation in the study were: age 65 years or older; no antibiotic, antimycotic or probiotic therapy in the previous 6 months; no history of hospitalization or diarrhea in the previous 6 months. All study participants gave general written informed consent prior to admission to hospital including molecular analysis of blood, biopsy and stool samples for scientific reasons. An additional ethical vote for intestinal microbiota analysis from stool samples was not deemed necessary by the local ethical board of the Medical Faculty of the Christian-Albrechts-University (CAU) in Kiel. Fecal samples were stored at −80°C until further use.

### Tracking of *Clostridium difficile* in fecal samples

Because the production of toxin A and B by *C. difficile* strains is associated with CDAD, all fecal samples were screened for presence of these toxins as well as for *glutamate dehydrogenase* antigen with an enzyme-linked immunosorbent assay (ELISA) at the Institute of Infectious Diseases (UKSH, Campus Kiel). *C. difficile* positivity of stool samples was verified by detection of the specific *C. difficile* toxin A and B, with a membrane immunoassay (TECHLAB; CLOSTRIDIUM DIFFICILE QUIK CHEK COMPLETE KIT, Alere GmbH, Köln, Germany).

### Total DNA and RNA extraction

Total DNA extraction was performed with incubation of fecal samples in 200 µl TL-buffer and 25 µl proteinase-K for two hours at 56°C, before using the FastDNASPIN KIT FOR SOIL (Qiagen, Hilden, Germany) as previously described [42]. Total RNA from feces was isolated using the PowerMicrobiome RNA Isolation Kit (MO BIO Laboratories Inc, Carlsbad, CA) following the manufacturer's protocol. Extracted RNA was reverse transcribed into cDNA with the High Capacity cDNA Reverse Transcription Kit (Applied Biosystems, Foster City, USA) according to the manufacturer's instructions.

### PCR amplification of variable region V1 to V2

The variable regions V1 and V2 of the 16S rRNA were amplified. Samples preparation and high throughput sequencing were performed according to previous studies [43].

### Data cleansing and statistics

Data were preprocessed using MOTHUR version 1.27 [44] as described before [43]. Data analysis and multivariate statistical approaches were done in R v3.0.0 (http://www.R-project.org/). Analyses were separately performed with DNA-and RNA-level data. P-values in multiple testing scenarios were subjected to Benjamini-Hochberg correction (“q-values”) [45]. Beta diversity analysis was conducted via redundancy analysis (RDA) [46] as implemented in the R package vegan v2.0-7 [47]. Count data of operational taxonomic units (OTU; defined by 97% sequence similarity) were Hellinger-transformed in order to produce valid results in RDA [48]. Alpha diversity was described by effective OTU richness ^1^D (known as the Shannon numbers equivalent) [49], [50], taking both the numbers and relative abundances of OTUs into account. The ^1^D values were Box-Cox transformed to meet homoscedasticity and normality of residuals in subsequent analysis of variance (ANOVA).

In a first step, the effect of *C. difficile* associated diarrhea development in connection with β-lactam AB classes cephalosporins and Ampicillin/Sulbactam was assessed by a 2×2 factorial ANOVA. A subsequent one-way ANOVA was conducted with only AB as the independent factor, including also quinolones as well as the control group. Data from *C. difficile*-positive individuals were included in the second ANOVA if the *C. difficile* infection effect was non-significant in the first one. With significance of the second ANOVA, pairwise comparisons of factor levels were conducted.

In beta diversity, OTUs most likely to be responsible for differences between the group of AB treated individuals and controls were determined. OTUs were pre-filtered by profile likelihood selection [51] according to their vector lengths calculated from RDA scores (scaling 1). Pre-filtered OTUs significantly correlated with axes in the RDA model were subjected to indicator analysis with the R package indicspecies v1.6.7 [52].

## Supporting Information

Figure S1 Distribution of Shannon number equivalents at RNA level in healthy controls and during treatment with Cephalosporins/Ampicillin/Sulbactam and Fluoroquinolones.
(PDF)

Figure S2 Distribution of OTUs at bacterial phylum level in fecal samples of healthy controls and during antibiotic treatment with Ampicillin/Sulbactam, Cephalosporins and Fluoroquinolones in CDAD positive and CDAD negative individuals at DNA and RNA level.
(PDF)

Figure S3 Distribution of OTUs at bacterial family level in fecal samples of healthy controls and during antibiotic treatment with Ampicillin/Sulbactam, Cephalosporins and Fluoroquinolones in CDAD positive and CDAD negative individuals at DNA and RNA level.
(PDF)

Table S1 **a:** Indicator OTUs of controls and AB treatment at the DNA level (Ctrl - controls, Fluoro-quinol - Fluoroquinolones). **b:** Indicator OTUs of controls and AB treatment at the RNA level (Ctrl - controls; AB includes Ampicillin/Sulbactam, Cephalosporins and fluoroquinolone). **c:** Indicator OTUs of individuals with and without CDAD at RNA and DNA level after treatment with Ampicillin/Sulbactam and cephalosporins.
(DOCX)

Table S2 Characteristics of study participants.
(DOCX)
