# Supplementary figures and images for: Effects of β-Lactam Antibiotics and Fluoroquinolones on Human Gut Microbiota in Relation to Clostridium difficile Associated Diarrhea
Source: PLoS One. 2014 Feb 28;9(2):e89417. doi: 10.1371/journal.pone.0089417 (PMC3938479; doi:10.1371/journal.pone.0089417)

Figure S1

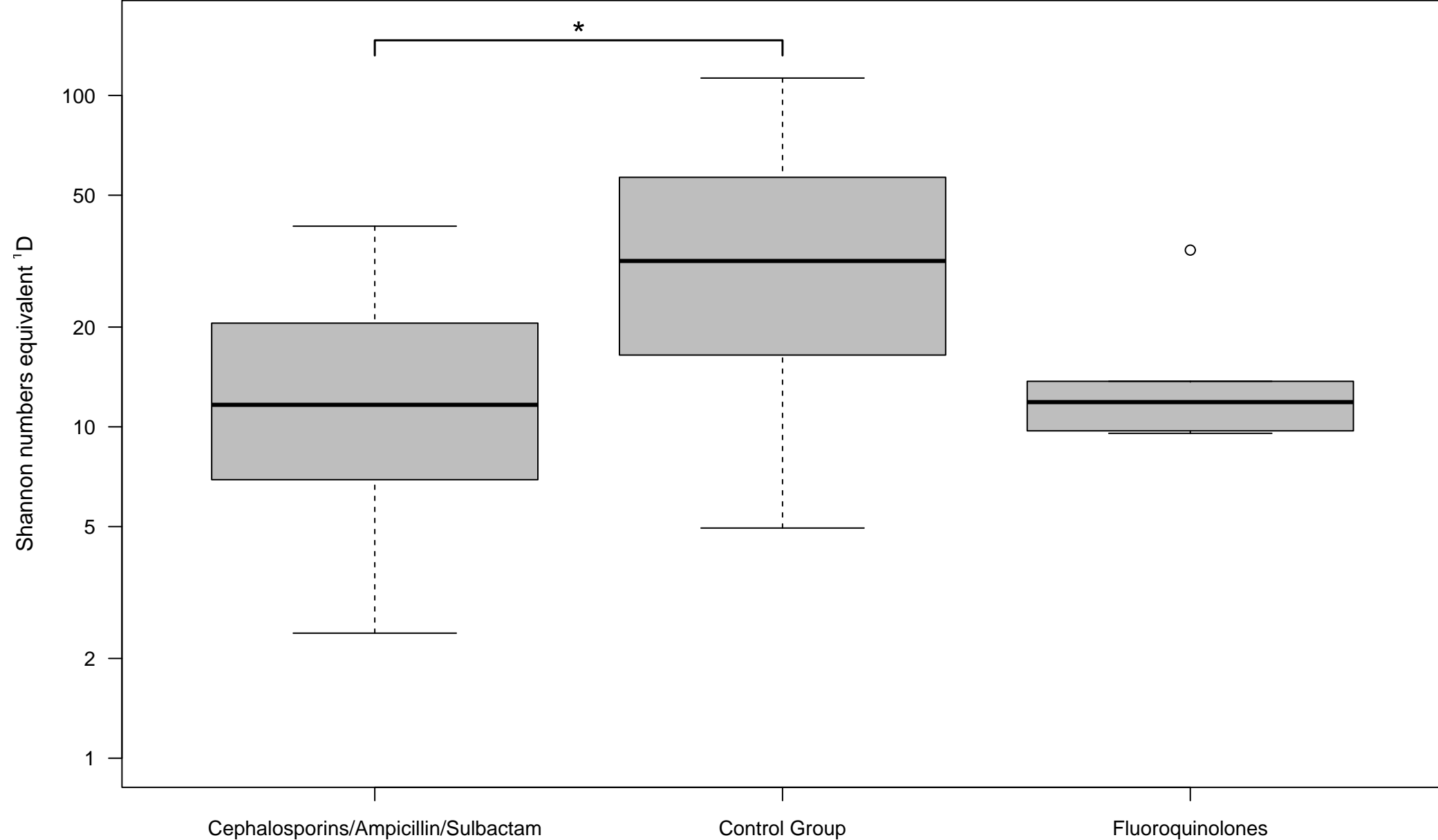

Supplement: Figure S1 — Distribution of Shannon number equivalents at RNA level in healthy controls and during treatment with Cephalosporins/Ampicillin/Sulbactam and Fluoroquinolones. (PDF) [file pone.0089417.s001.pdf]

Figure S2

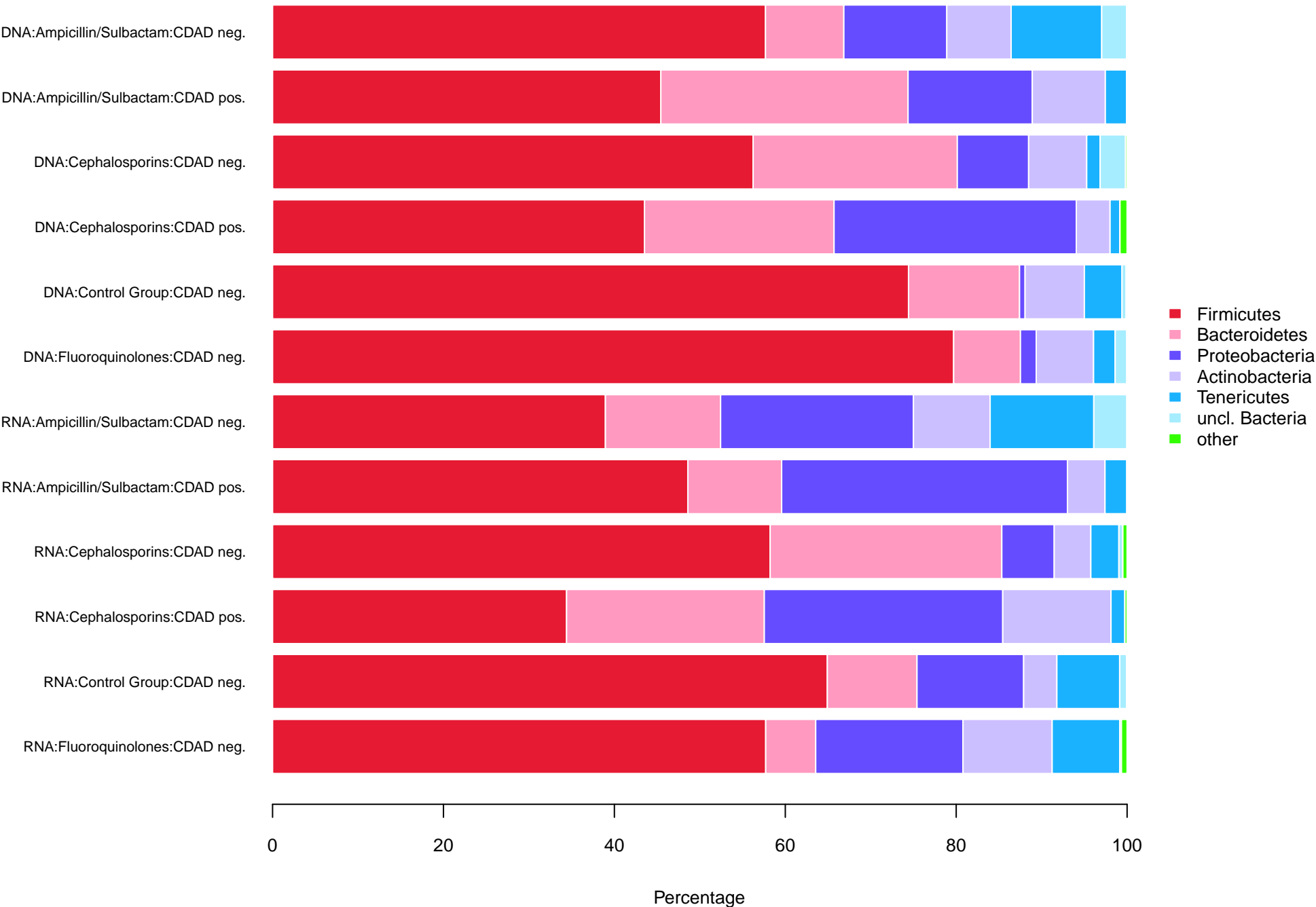

Supplement: Figure S2 — Distribution of OTUs at bacterial phylum level in fecal samples of healthy controls and during antibiotic treatment with Ampicillin/Sulbactam, Cephalosporins and Fluoroquinolones in CDAD positive and CDAD negative individuals at DNA and RNA level. (PDF) [file pone.0089417.s002.pdf]

Figure S3

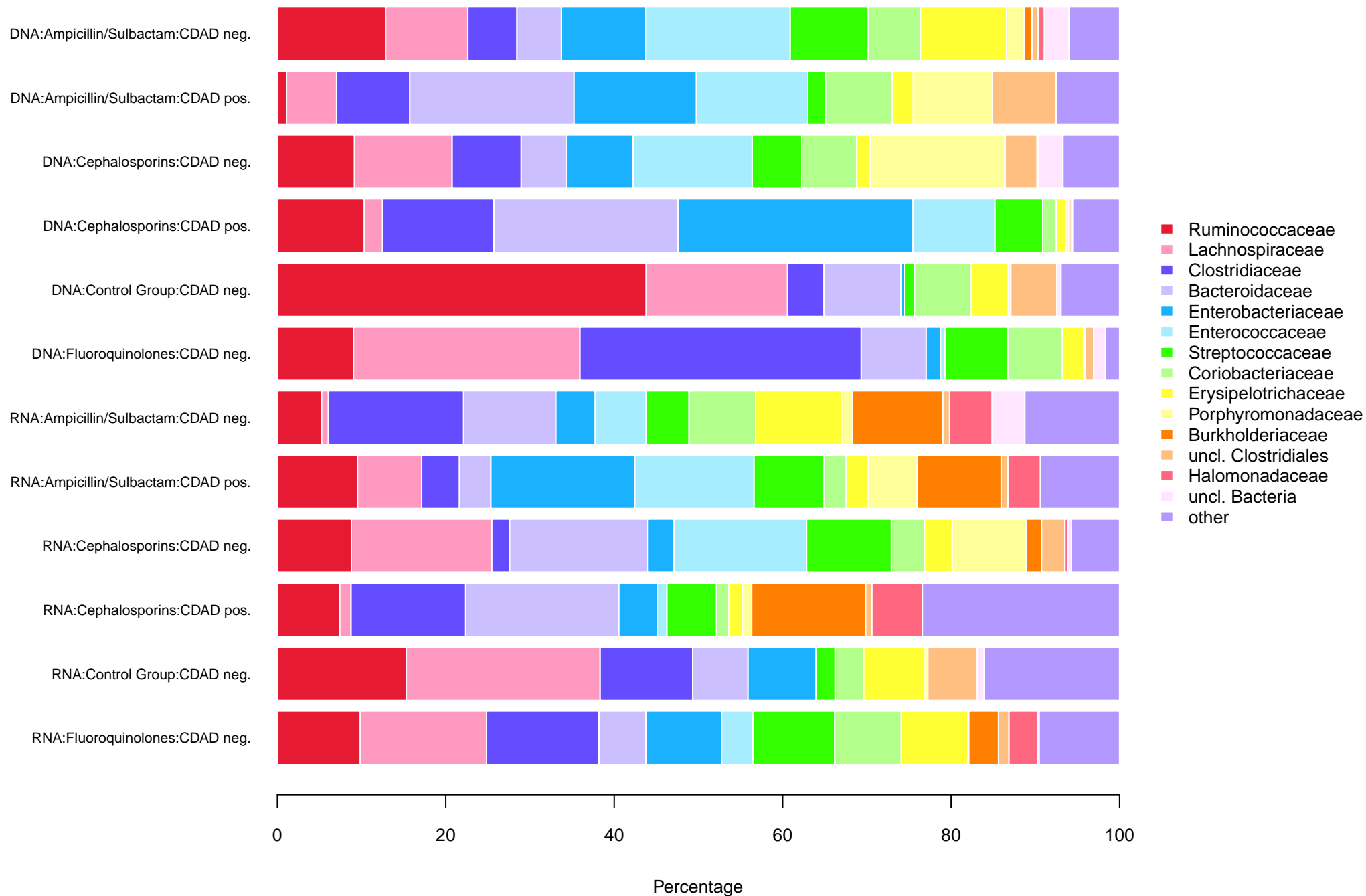

Supplement: Figure S3 — Distribution of OTUs at bacterial family level in fecal samples of healthy controls and during antibiotic treatment with Ampicillin/Sulbactam, Cephalosporins and Fluoroquinolones in CDAD positive and CDAD negative individuals at DNA and RNA level. (PDF) [file pone.0089417.s003.pdf]
