# Supplementary material for: Effects of β-Lactam Antibiotics and Fluoroquinolones on Human Gut Microbiota in Relation to Clostridium difficile Associated Diarrhea
Source: PLoS One. 2014 Feb 28;9(2):e89417. doi: 10.1371/journal.pone.0089417 (PMC3938479; doi:10.1371/journal.pone.0089417)
Supplement: Table S1 — a: Indicator OTUs of controls and AB treatment at the DNA level (Ctrl - controls, Fluoro-quinol - Fluoroquinolones). b: Indicator OTUs of controls and AB treatment at the RNA level (Ctrl - controls; AB includes Ampicillin/Sulbactam, Cephalosporins and fluoroquinolone). c: Indicator OTUs of individuals with and without CDAD at RNA and DNA level after treatment with Ampicillin/Sulbactam and cephalosporins. (DOCX) [file pone.0089417.s004.docx]

**Table S1a**

| **OTU** | **Taxonomy** | | **Indicator** | **DNA** | **DNA** |
| --- | --- | --- | --- | --- | --- |
|  | **Family/Genus** | **Species** | **DNA level** | **p-value** | **q-value** |
| 238 | *Faecalibacterium* | *prausnitzii* | control | <0.001 | < 0.001 |
| 239 | *Blautia* | uncl. | Fluoroquinolon | 0.002 | 0.008 |
| 240 | *Coprococcus* | uncl. | control | 0.03 | 0.04 |
| 241 | *Faecalibacterium* | *prausnitzii* | control | <0.001 | < 0.001 |
| 242 | *Faecalibacterium* | *prausnitzii* | control | 0.02 | 0.03 |
| 244 | *Blautia* | uncl. | Fluoroquinolon | 0.01 | 0.03 |
| 247 | *Coprococcus* | uncl. | control | 0.01 | 0.03 |
| 249 | *Eubacterium* | uncl. | control | 0.01 | 0.03 |
| 250 | *Subdoligranulum* | uncl. | Fluoroquinolon | 0.01 | 0.03 |
| 254 | *Blautia* | uncl. | Fluoroquinolon | 0.018 | 0.03 |
| 274 | *Ruminococcaceae* | uncl. | Fluoroquinolon | 0.001 | 0.008 |
| 279 | *Blautia* | uncl. | Fluoroquinolon | 0.004 | 0.01 |
| 282 | Bacteria | uncl. | Fluoroquinolon | 0.01 | 0.03 |
| 287 | *Ruminococcus* | uncl. | Fluoroquinolon | 0.002 | 0.008 |
| 4107 | *Enterobacter* | uncl*.* | ß-lactam | 0.01 | 0.03 |
| 4208 | Clostridium | uncl. | Fluoroquinolon | 0.002 | 0.008 |
| 4289 | *Enterococcus* | *lacti* | ß-lactam | 0.002 | 0.008 |
| 4330 | *Adlercreutzia* | uncl. | Fluoroquinolon | 0.02 | 0.04 |

**Table S1b**

| **OTU** | **Taxonomy** | | **Indicator** | **RNA** | **RNA** |
| --- | --- | --- | --- | --- | --- |
|  | **Family/Genus** | **Species** | **RNA level** | **p-value** | **q-value** |
| 237 | *Shewanella* | *algae* | AB | <0.001 | <0.001 |
| 238 | *Faecalibacterium* | *prausnitzii* | Ctrl | 0.001 | 0.002 |
| 240 | *Coprococcus* | uncl. | Ctrl | 0.001 | 0.001 |
| 241 | *Faecalibacterium* | *prausnitzii* | Ctrl | <0.001 | 0.001 |
| 249 | *Eubacterium* | uncl. | Ctrl | <0.001 | <0.001 |
| 256 | *Eubacterium* | uncl. | Ctrl | 0.0003 | 0.001 |
| 261 | *Lachnospiraceae* | uncl. | Ctrl | <0.001 | <0.001 |
| 4141 | *Streptococcus* | *themophilus* | AB | 0.2 | 0.032 |
| 4205 | *Clostridium* | *ramosum* | AB | <0.001 | <0.001 |
| 4206 | *Clostridium* | uncl. | Ctrl | 0.003 | <0.001 |
| 4289 | *Enterococcus* | lacti | AB | 0.001 | <0.001 |
| 4329 | *Eggerthella* | *lenta* | AB | 0.012 | 0.023 |
| 4428 | *Clostridium* | uncl. | Ctrl | <0.001 | <0.000 |
| 4575 | *Halomonas* | uncl. | AB | <0.001 | <0.000 |
| 4576 | *Halomonas* | uncl. | AB | <0.001 | <0.000 |
| 4602 | *Ralstonia* | uncl. | AB | <0.001 | <0.000 |
| 4766 | *Propionibacterium* | *acne* | AB | 0.002 | 0.004 |
| 4854 | *Staphylococcus* | *epidermidis* | AB | 0.02 | 0.032 |
| 4902 | Granulicatella | *adiacens* | AB | 0.001 | 0.003 |
| 4903 | Granulicatella | uncl. | AB | 0.014 | 0.025 |

**Table S1c**

| **OTU** | **Taxonomy** | | **Indicator level** | **Group** |
| --- | --- | --- | --- | --- |
|  | **Family/Genus** | **Species** |  | |
| 4144 | *Streptococcus* | *pseudopneumonia* | RNA | CDAD |
| 4330 | *Adlercreutzia* | uncl. | DNA | Non-CDAD |
| 4327 | *Collinsella* | *aerofaciens* | DNA | Non-CDAD |
| 4429 | *Clostridium* | *difficile* | DNA | CDAD |
| 4552 | *Enhydrobacter* | *aerosaccus* | RNA | CDAD |
| 4902 | *Granulicatella* | adiacens | RNA | CDAD |
