# Supplementary material for: Effects of β-Lactam Antibiotics and Fluoroquinolones on Human Gut Microbiota in Relation to Clostridium difficile Associated Diarrhea
Source: PLoS One. 2014 Feb 28;9(2):e89417. doi: 10.1371/journal.pone.0089417 (PMC3938479; doi:10.1371/journal.pone.0089417)
Supplement: Table S2 — Characteristics of study participants. (DOCX) [file pone.0089417.s005.docx]

| **Group** | **Individual** | **Age**  **(mean +/- SD)** | **Gender** | **AB** |
| --- | --- | --- | --- | --- |
| Healthy controls | 1-10 | (71.2 +/- 4.5) | Male | **/** |
|  | 11-18 | (70.3 +/- 3.5) | Female | **/** |
| CDAD negative during therapy with Ampicillins |  |  |  |  |
|  | 1 | 87 | Female | Ampicillin/Sulbcatam |
|  | 2 | 72 | Male | Ampicillin/Sulbcatam |
|  | 3 | 69 | Male | Ampicillin/Sulbcatam |
|  | 4 | 76 | Female | Ampicillin/Sulbcatam |
|  | 5 | 72 | Male | Ampicillin/Sulbcatam |
| CDAD negative during therapy with cephalosporins |  |  |  |  |
|  | 1 | 74 | Female | Cefazolin |
|  | 2 | 73 | Male | Ceftazidim |
|  | 3 | 73 | Female | Ceftriaxon |
|  | 4 | 78 | Female | Ceftriaxon |
|  | 5 | 69 | Male | Ceftazidim |
| CDAD negative during therapy with fluoroquinolones |  |  |  |  |
|  | 1 | 65 | Female | Ciprofloxacin |
|  | 2 | 75 | Female | Moxifloxacin |
|  | 3 | 91 | Female | Ciprofloxacin |
|  | 4 | 72 | Male | Moxifloxacin |
|  | 5 | 67 | Female | Ciprofloxacin |
| CDAD **positive** during therapy with Ampicillins |  |  |  |  |
|  | 1 | 67 | Female | Ampicillin/Sulbcatam |
|  | 2 | 81 | Male | Ampicillin/Sulbcatam |
|  | 3 | 76 | Female | Ampicillin/Sulbcatam |
|  | 4 | 69 | Female | Ampicillin/Sulbcatam |
|  | 5 | 72 | Male | Ampicillin/Sulbcatam |
| CDAD **positive** during therapy with cephalosporins |  |  |  |  |
|  | 1 | 82 | Male | Ceftazidim |
|  | 2 | 76 | Female | Ceftriaxon |
|  | 3 | 77 | Female | Ceftriaxon |
|  | 4 | 69 | Male | Ceftazidim |
|  | 5 | 72 | Female | Ceftriaxon |

**Table S2**
